# Revisiting the Transcriptome Landscape of Pig Embryo Implantation Site at Single-Cell Resolution

**DOI:** 10.3389/fcell.2022.796358

**Published:** 2022-05-04

**Authors:** Qing Tian, Jia-Peng He, Can Zhu, Qiu-Yang Zhu, Yu-Gu Li, Ji-Long Liu

**Affiliations:** Guangdong Laboratory for Lingnan Modern Agriculture, College of Veterinary Medicine, South China Agricultural University, Guangzhou, China

**Keywords:** embryo implantation, pig, endometrium, single-cell RNA-seq, intercellular crosstalk

## Abstract

Litter size is one of the most economically important traits in commercial pig farming. It has been estimated that approximately 30% of porcine embryos are lost during the peri-implantation period. Despite rapid advances over recent years, the molecular mechanism underlying embryo implantation in pigs remains poorly understood. In this study, the conceptus together with a small amount of its surrounding endometrial tissues at the implantation site was collected and subjected to single-cell RNA-seq using the 10x platform. Because embryo and maternal endometrium were genetically different, we successfully dissected embryonic cells from maternal endometrial cells in the data according to single nucleotide polymorphism information captured by single-cell RNA-seq. Undoubtedly, the interaction between trophoblast cells and uterine epithelial cells represents the key mechanism of embryo implantation. Using the CellChat tool, we revealed cell-cell communications between these 2 cell types in terms of secreted signaling, ECM-receptor interaction and cell-cell contact. Additionally, by analyzing the non-pregnant endometrium as control, we were able to identify global gene expression changes associated with embryo implantation in each cell type. Our data provide a valuable resource for deciphering the molecular mechanism of embryo implantation in pigs.

## 1 Introduction

Litter size is one of the most economically important traits in commercial pig farming. It has been estimated that approximately 30% of porcine embryos are lost between days 12 and 30 of pregnancy, i.e., during the peri-implantation period (Tayade et al., 2006; Zang et al., 2021b). Embryo implantation in the pig is unique in that a typical non-invasive central-type is employed, which is characterized by a lengthy pre-attachment period with a rapid transformation in embryo morphology from ovoid to a filamentous shape (Lee and DeMayo 2004). Embryo implantation occurs on day 15 of pregnancy, which initiates epitheliochorial placentation (Bazer and Johnson 2014). So far, the molecular mechanism underlying embryo implantation in pigs remains poorly understood.

Previously, global gene expression changes in the porcine endometrium during the peri-implantation period have been determined by using microarrays and RNA-Seq (Ostrup et al., 2010; Samborski et al., 2013a; Samborski et al., 2013b; Franczak et al., 2013; Gu et al., 2014; Chen et al., 2015; Huang et al., 2015; Lin et al., 2015). The endometrium is a complex tissue consisting of many cell types, including luminal and glandular epithelial cells, stromal cells, endothelial cells, and various immune cells. Thus, these studies were unable to accurately capture cell-type-specific gene expression changes. Recently, laser capture microdissection (LCM) has been applied to obtain the global gene expression profiles in luminal epithelial cells, glandular epithelial cells and stromal cells of peri-implantation porcine endometrium (Zeng et al., 2018; Zeng et al., 2019; Wang et al., 2021). The limitation of LCM-based methods is that cell types such as various immune cells can be hardly isolated. In theory, fluorescence-activated cell sorting (FACS) might be suitable to purify any endometrial cell types (Stas et al., 2020). However, the lack of sophisticated antibodies to sort different cell types is the limiting factor, since reliable cell-type-specific cell-surface protein markers are yet to be discovered.

Single-cell RNA-seq, which allows large-scale transcript profiling for thousands of cells in a single experiment, is a highly accurate tool for quantifying gene expression in a highly heterogeneous tissue (Svensson et al., 2018). In contrast to the conventional RNA-seq, single-cell RNA-seq has several advantages. Firstly, it is an unbiased method which is not limited to detecting known cell types. Secondly, it provides individual transcriptomes for each cell, instead of merely a cell-averaged transcriptome. Lastly, intercellular cross-talk between cell types can be inferred based on the expression of ligand-receptor pairs. In the present study, we took advantage of the single-cell RNA-seq approach to investigate the global gene expression changes in the porcine uterus and conceptus during embryo implantation. Our study contributes to an increase in the knowledge on molecular mechanisms underlying embryo implantation in pigs.

## 2 Materials and Methods

### 2.1 Sample Collection

Adult Bama mini-pigs were obtained from the Kangtai Pasture Co. Ltd. (Yangjiang, China). The gilts of similar age and weight were observed twice a day for estrous behavior by using intact boars. Gilts were mated to boars on the day of first standing estrus and again 24 h later. The first day of mating was considered to be day 0 of gestation. The whole uterus was obtained from gilts slaughtered on day 15 of pregnancy. Each uterus was cut into 10-cm segments. For each gilt, half of the uterine segments were flushed with phosphate-buffered saline (PBS) and pregnancy was confirmed by the presence of normal conceptus in the uterine flushing. If pregnancy was confirmed, the other half of intact uterine segments were opened longitudinally at the anti-mesometrial site and the implantation site (including conceptus and the endometrial tissue beneath it) was collected under dissecting microscope magnification. Approximately 200 mg tissues were recovered for each implantation site. If pregnancy was not confirmed, endometrial tissues were randomly collected from the mesometrial side in a similar way, serving as control. All animal procedures were approved by the Institutional Animal Care and Use Committee of South China Agricultural University (No. 2020B078, approved on 29/09/2020).

### 2.2 Single-Cell Dissociation

Single-cell dissociation was performed as described previously (Yang et al., 2021a; He et al., 2021). Endometrial tissues and conceptus fragments from 3 gilts for each group were minced with a blade and then incubated in dissociation buffer containing 2 mg/ml Collagenase II (#C6885, Sigma-Aldrich), 10 mg/ml Dispase II (#354235, Corning) and 50,000 U/ml DNase I (#DN25, Sigma-Aldrich) for up to 30 min at 37°C in a shaking incubator. The digestion progress was monitored with a microscope until a single cell suspension was achieved. To remove undigested tissues, the single-cell suspension was then passed through a 40-μm cell strainer and cells were spun down at 250 g at 4°C for 4 min. Red blood cells (RBC) were removed by using RBC Lysis Buffer (#00-4333, Invitrogen). Cell viability was measured by AO/PI solution (#CS2-0106, Nexcelom Bioscience). The quality control criteria for single-cell suspension were cell viability > 80% and the percentage of cell clumps < 10%.

### 2.3 Single-Cell RNA-Seq Library Preparation and Sequencing

The final concentration of single-cell suspension was adjusted to 1,000 cells/μl. In order to recover 8,000-10,000 cells, a volume of 15 µl was loaded into one channel of the Chromium™ Single Cell B Chip (#1000073, 10x Genomics). Single-cell bar-coding, cDNA synthesis and library preparation were performed by using the Chromium Single Cell 3′ Library & Gel Bead Kit v3 (#1000075, 10x Genomics). DNA libraries were then sequenced on an Illumina novaseq 6000 system configured with the paired-end 150-bp protocol for a sequencing depth of approximately 400 million reads per library.

### 2.4 Single-Cell RNA-Seq Data Processing

Raw data of fastq files were aligned to the Sscrofa11.1 pig reference genome by using the CellRanger software v3.0.1 (10x Genomics). The resulting gene counts matrix was processed with a computational pipeline as described previously (Yang et al., 2021a; He et al., 2021). Briefly, cells with <200 or >6000 unique genes, as well as cells with >25% of mitochondrial counts, were discarded. On the other hand, genes expressed in <3 cells were removed. The filtered gene count matrix was then normalized, scaled and subjected to dimensional reduction. The cell type label for each cell cluster was manually assigned based on canonical cell markers. The Wilcoxon rank-sum test was used to identify differentially expressed genes in the same cell type between groups with min.logfc being set to 0.25 and min.pct being set to 0.20.

### 2.5 Dissecting Embryonic Cells From Maternal Uterine Cells in the Data

Embryonic cells were dissected from maternal uterine cells according to single-nucleotide polymorphisms (SNPs) captured by single-cell RNA-seq. Briefly, reads were re-mapped to the reference genome with minimap2 (Li 2018) and SNP calls were then used to predict the origin of cells by using Souporcell v2 (Heaton et al., 2020).

### 2.6 Gene Ontology Analysis

Gene ontology (GO) analysis was based on the biological process category defined in the Mouse Genome Informatics (MGI) GOslim database (Law and Shaw 2018). The hypergeometric distribution was employed for enrichment test as described previously (Liu et al., 2016). *p* < 0.05 was considered statistically significant.

### 2.7 Pathway Enrichment Analysis

Pathway enrichment analysis was performed by using the Metascape v7.4 software (Zhou et al., 2019). The significance threshold for false discovery rate (FDR) was set at 0.05.

### 2.8 Cell-Cell Communication

The R package CellChat v1.1.0 (Jin et al., 2021) was used to infer cell-cell communications based on ligand-receptor interactions as described previously (He et al., 2021). *p* < 0.05 were considered significant.

## 3 Results

### 3.1 A Single-Cell Atlas of Pig Embryo Implantation Site

**FIGURE 1:**
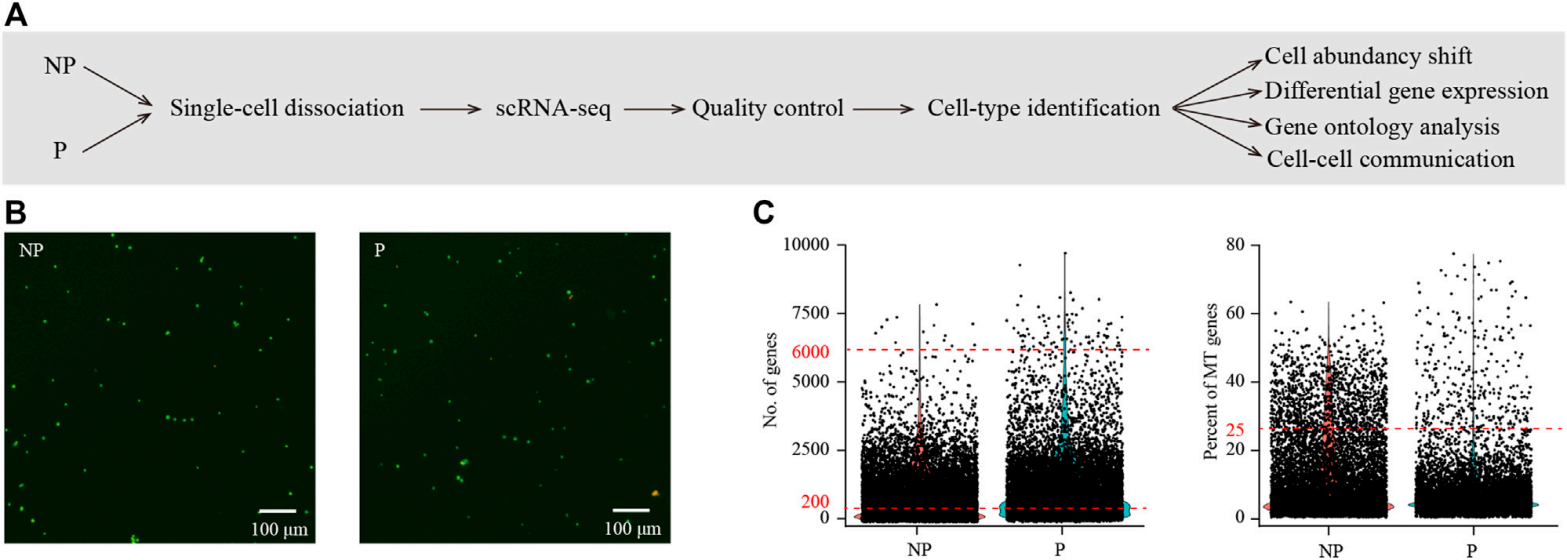
Single-cell transcriptome analysis of the implantation site in porcine uterus on day 15 of pregnancy. **(A)** A flowchart overview of this study. NP, non-pregnant uterus, served as control; P, pregnant uterus (the implantation site of pregnant uterus). **(B)** Cell viability analysis of single-cell suspension. Cells were stained with the AO/PI solution. Cells in green were live and cells in yellow were dead. **(C)** Single-cell RNA-seq data pre-processing and quality control. Cells with detected genes of fewer than 200 or more than 6000 were removed (left) and only cells with total mitochondrial gene expression below 25% were kept (right).

In order to generate cell-type resolved map of the pig embryo implantation site, we performed single-cell RNA-seq analysis (Figure 1A). The conceptus together with a small amount of its surrounding endometrial tissues at the implantation site was collected from pregnant pigs. To serve as control, endometrial tissues from paralleled non-pregnant pigs were randomly collected from the mesometrial side of uterus where embryo implantation was expected to take place. Both samples from the implantation site of pregnant pigs (P) and control samples from non-pregnant pigs (NP) were subjected to single-cell dissociation (Figure 1B). Single-cell RNA-seq data were generated by using the 10x Genomics platform. To avoid batch effect, all samples were processed and sequenced in parallel. After quality control, a total of 12,415 cells (7746 for P and 4669 for NP) were obtained (Figure 1C).

**FIGURE 2:**
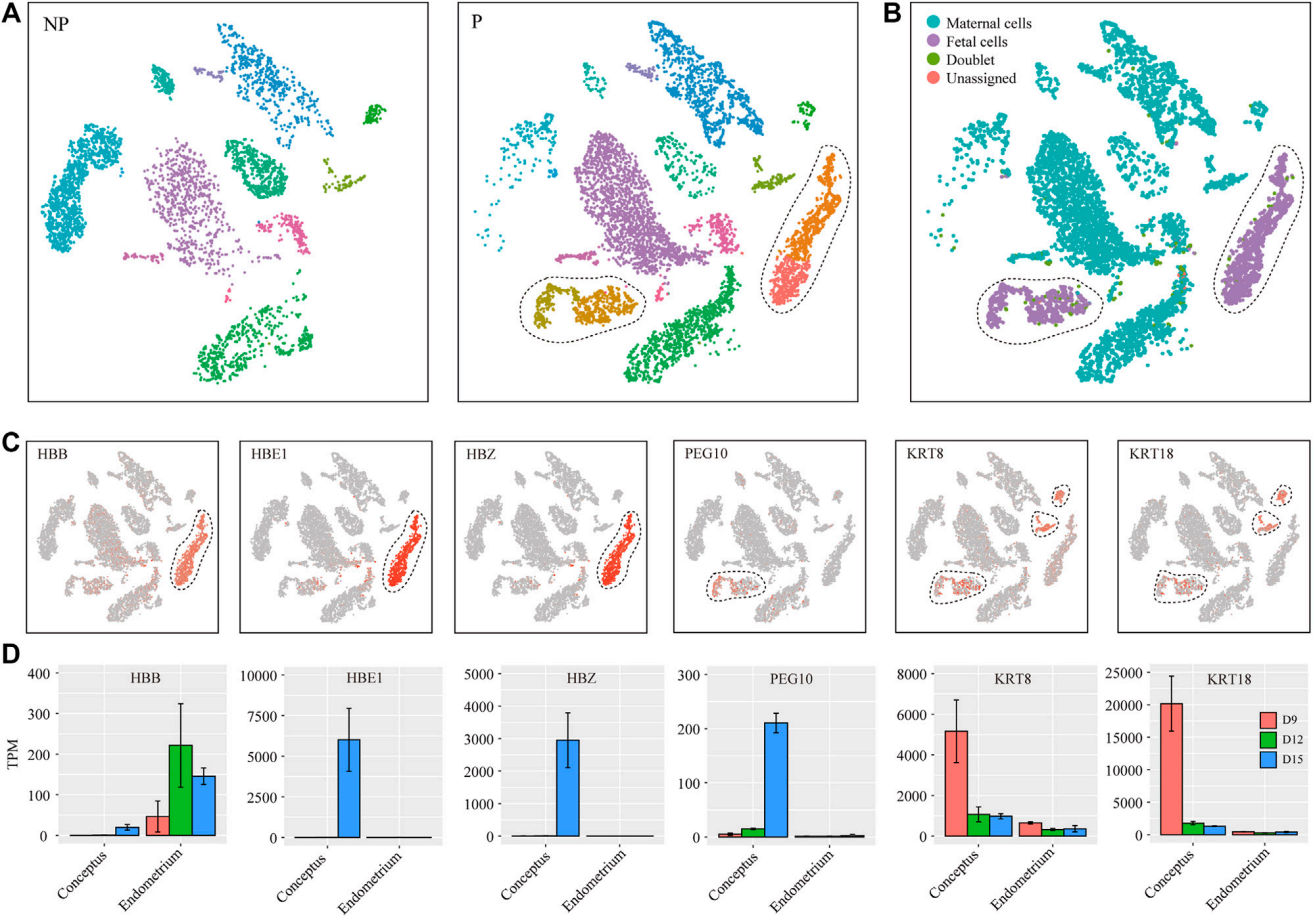
Dissecting fetal cells from maternal cells in single-cell RNA-seq data. **(A)** The t-Stochastic Neighbor Embedding (tSNE) representation of single-cell RNA-seq data obtained from NP and P of porcine uterus. Dashed lines denote the boundaries of fetal cells in P which were absent in NP. **(B)** The cell type annotation by the Souporcell software. Maternal cells, fetal cells, doublets and unassigned cells were displayed in tSNE plot. **(C)** The expression pattern of canonical marker genes projected onto tSNE plots. Shown were marker genes for erythroid cells and trophoblast cells. Dashed lines denoted the boundaries of the cell cluster of interest. **(D)** Validation of marker genes using public bulk-tissue datasets. RNA-seq data for Yorkshire pig conceptus (PRJNA646603) and endometrium (PRJNA393735) from days 9, 12 and 15 of pregnancy were downloaded from Sequence Read Archive (SRA) database. Gene expression levels were normalized as transcript per million (TPM).

**FIGURE 3:**
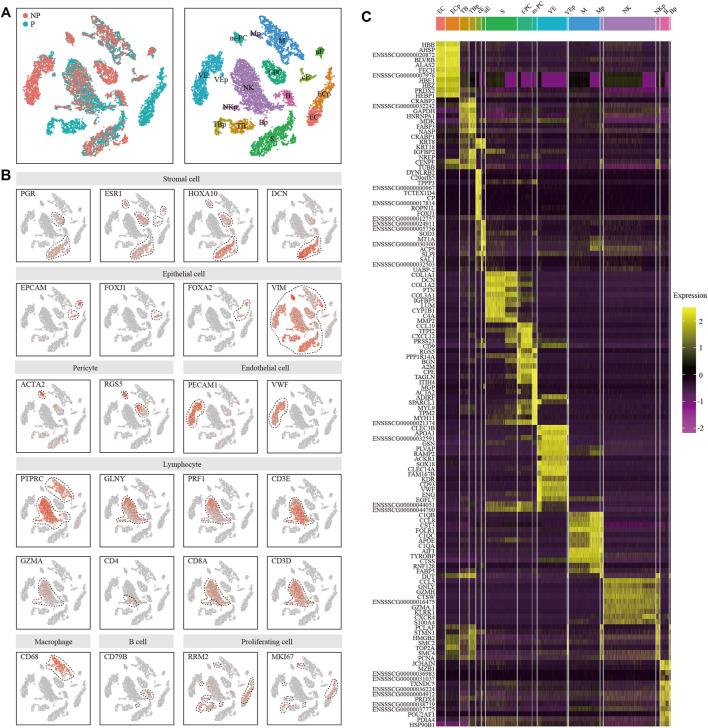
Identification of different cell types in porcine uterus by using canonical marker genes. **(A)** The tSNE representation of single-cell RNA-seq data obtained from NP and P of porcine uterus. Single cells were grouped by cellular origin (right) and cell clusters (left). EC, erythroid cells, ECp, proliferating erythroid cells; TB, trophoblast cells, TBp, proliferating trophoblast cells; cE, ciliated epithelial cells; uE, unciliated epithelial cells; S, stromal cells; f-PC, fibroblast-like pericytes; m-PC, smooth muscle-like pericytes; VE, vascular endothelial cells; VEp, proliferating vascular endothelial cells; M, macrophages; Mp, proliferating macrophages; NK, natural killer cells; NKp, proliferating natural killer cells; B, B cells; Bp, proliferating B cells. **(B)** The expression pattern of canonical marker genes projected onto tSNE plots. Shown were marker genes for stromal cells, epithelial cells, pericytes, endothelial cells, lymphocytes, macrophages, B cells and proliferating cells. Dashed lines denote the boundaries of the cell cluster of interest. **(C)** Heatmap of top 10 gene expression signatures for each cell type.

We performed graph-based clustering of the single-cell RNA-seq data. The challenge of this study was to dissect embryonic cells from maternal uterine cells. Using NP as control, we found 4 cell clusters that were unique for P (Figure 2A). We suspected that these 4 cell clusters were likely embryo-derived. Embryonic cell and maternal uterine cells were genetically different and many single-nucleotide polymorphisms (SNPs) might be captured by single-cell RNA-seq. Therefore, we employed the Souporcell software to dissect embryonic cells from maternal uterine cells based on SNPs. In this way, we confirmed that these 4 cell clusters were indeed embryonic cells (Figure 2B). In order to further characterize these embryo-derived cells, we calculated their signature genes by using Wilcoxon rank sum test. We found that 2 cell clusters were erythroid cells expressing HBB, HBE1 and HBZ, and the other 2 cell clusters were trophoblast cells expressing PEG10, KRT8 and KRT18 (Figure 2C). Previously, bulk-tissue RNA-seq was performed on Yorkshire pig embryos (Zang et al., 2021a) and endometrial tissues (unpublished) from days 9, 12 and 15 of pregnancy. These bulk-tissue RNA-seq data provided validity of our findings by showing that HBE1 and HBZ were uniquely expressed in erythroid cells, while PEG10 was uniquely expressed in trophoblast cells (Figure 2D). Based on proliferation markers RRM2 and MKI67, erythroid cells could be divided into non-proliferating erythroid cells (EC) and proliferating erythroid cells (ECp). Similarly, trophoblast cells could be divided into non-proliferating trophoblast cells (TB) and proliferating trophoblast cells (TBp) (Figures 3A,B).

Besides these 4 embryo-derived cell clusters, we identified 13 maternal uterine cell clusters for NP and P combined (Figure 3A). Major cell types were defined by using the expression of known cell type-specific marker genes, with stromal cells expressing DCN (Sanches et al., 2010), epithelial cells expressing EPCAM (Jin 2019), pericytes expressing RGS5 (Mucenski et al., 2019), endothelial cells expressing PECAM1 and VWF (Kalucka et al., 2020), lymphocytes expressing PTPRC (Croy et al., 2012) (Figure 3B). Only 1 stromal cell cluster (S, PGR^+^ESR1^+^HOXA10^+^DCN^+^EPCAM^−^RGS5^-^) was found. There were 2 epithelial cell clusters, ciliated epithelial cells (cE, EPCAM^+^FOXJ1^+^) and unciliated epithelial cells (uE, EPCAM^+^FOXJ1^-^) (Fitzgerald et al., 2019; Wang et al., 2020). Two pericyte clusters, muscular pericytes (m-PC, RGS5^+^ACTA2^high^) and fibroblastic pericytes (f-PC, RGS5^+^ACTA2^low^), were identified (Shami et al., 2020). Vascular endothelial cells had 2 clusters, VE (PECAM1^+^VWF^+^MKI67^-^) and a proliferating subset VEp (PECAM1^+^VWF^+^MKI67^+^). There were 6 immune cell clusters including natural killer cells (NK, PTPRC^+^CD3E^+^), proliferating natural killer cells (NKp, PTPRC^+^CD3E^+^MKI67^+^), macrophages (M, PTPRC^+^CD68^+^), proliferating macrophages (M, PTPRC^+^CD68^+^MKI67^+^), B cells (B, PTPRC^+^CD79B^+^) and proliferating B cells (M, PTPRC^+^CD79B^+^MKI67^+^) (Yang et al., 2021a; He et al., 2021). Finally, we aimed to discover novel markers for each cell type. We selected genes that expressed significantly higher in the cell types of interest than the other cell types by Wilcoxon rank-sum test. A heatmap depicting the top 10 marker genes for each cell type is shown in Figure 3C. The complete lists of marker genes are presented in Supplementary Table S1.

### 3.2 Inferring Cell-Cell Communications at Embryo Implantation Site

**FIGURE 4:**
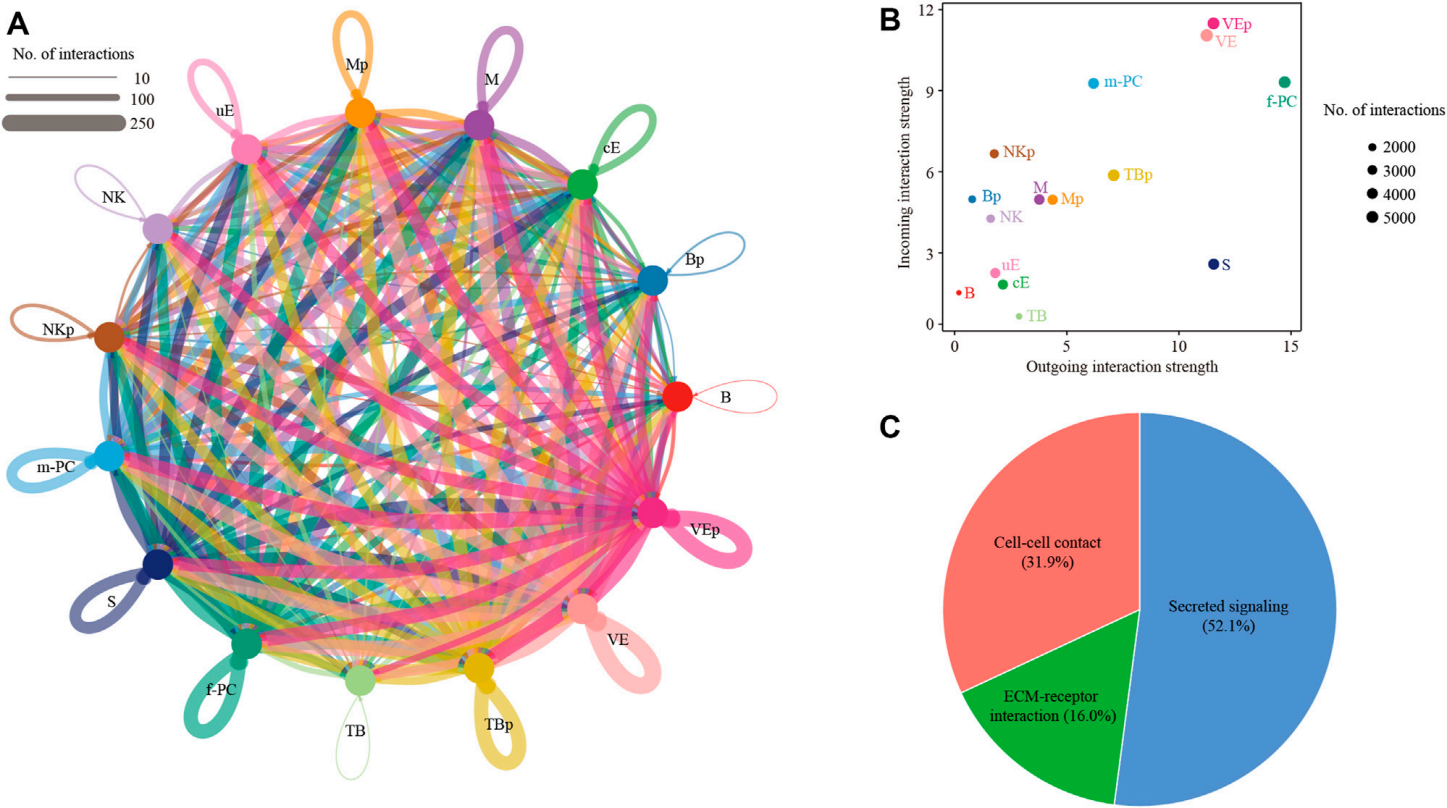
Cell-cell communication between different cell types. **(A)** Network plot showing ligand-receptor interactions underlying the cross-talk between different cell types. The number of interactions was indicated by the degree of thickness. **(B)** The incoming and outgoing interaction strength were defined by the CellChat software. The size of node represented the total number of interactions in each type of cells. **(C)** Pie chart showing the percentage of 3 categories of ligand-receptor interactions: cell-cell contact, ECM-receptor interaction and secreted signaling. TB, trophoblast cells, TBp, proliferating trophoblast cells; cE, ciliated epithelial cells; uE, unciliated epithelial cells; S, stromal cells; f-PC, fibroblast-like pericytes; m-PC, smooth muscle-like pericytes; VE, vascular endothelial cells; VEp, proliferating vascular endothelial cells; M, macrophages; Mp, proliferating macrophages; NK, natural killer cells; NKp, proliferating natural killer cells; B, B cells; Bp, proliferating B cells.

We reconstructed a cell-cell communication network between different cell types (Figure 4A). This network was based on 51,784 ligand-receptor interaction pairs inferred by the CellChat software (Jin et al., 2021). There were 3337, 3323, 4153, 4863, 4011, 5490, 5030, 3637, 3396, 2277, 2602, 1,393, 1921, 1,500 and 4851 ligand-receptor interaction pairs for cE, uE, S, f-PC, m-PC, VE, VEp, M, Mp, NK, NKp, B, Bp, TB and TBp, respectively (Figure 4B). These ligand-receptor interaction pairs could be further categorized as cell-cell contact (31.9%), ECM-receptor interaction (16.0%) and secreted signaling (52.1%) (Figure 4C).

**FIGURE 5:**
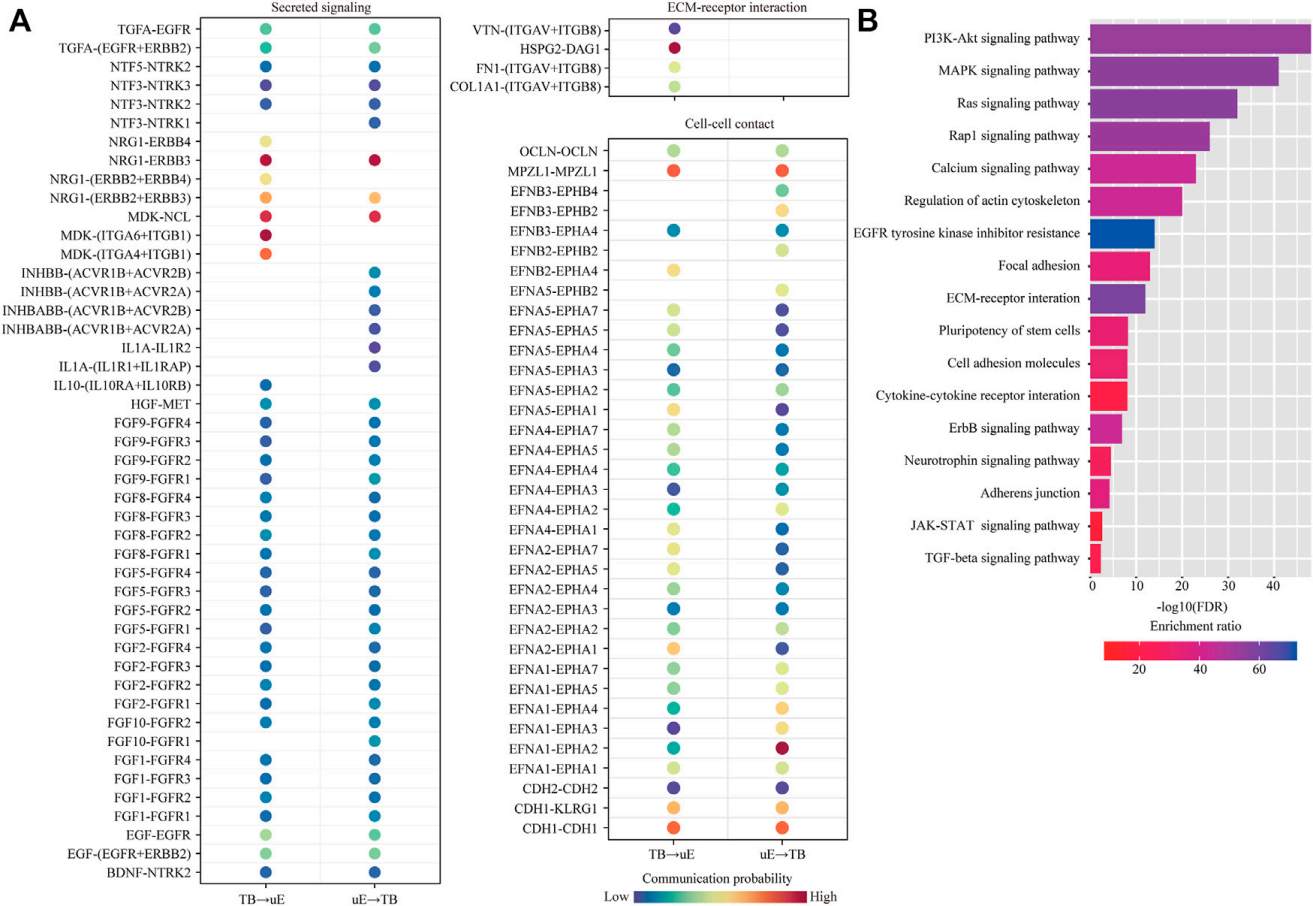
Cell-cell communication between unciliated epithelial cells and trophoblast cells. **(A)** Dot plot showing selected ligand-receptor interactions underlying the cross-talk between unciliated epithelial cells (uE) and trophoblast cells (TB). The communication probability defined by the CellChat software was indicated by color. **(B)** KEGG Pathway enrichment analysis of ligand-receptor pairs was performed by using the Metascape online tools.

**FIGURE 6:**
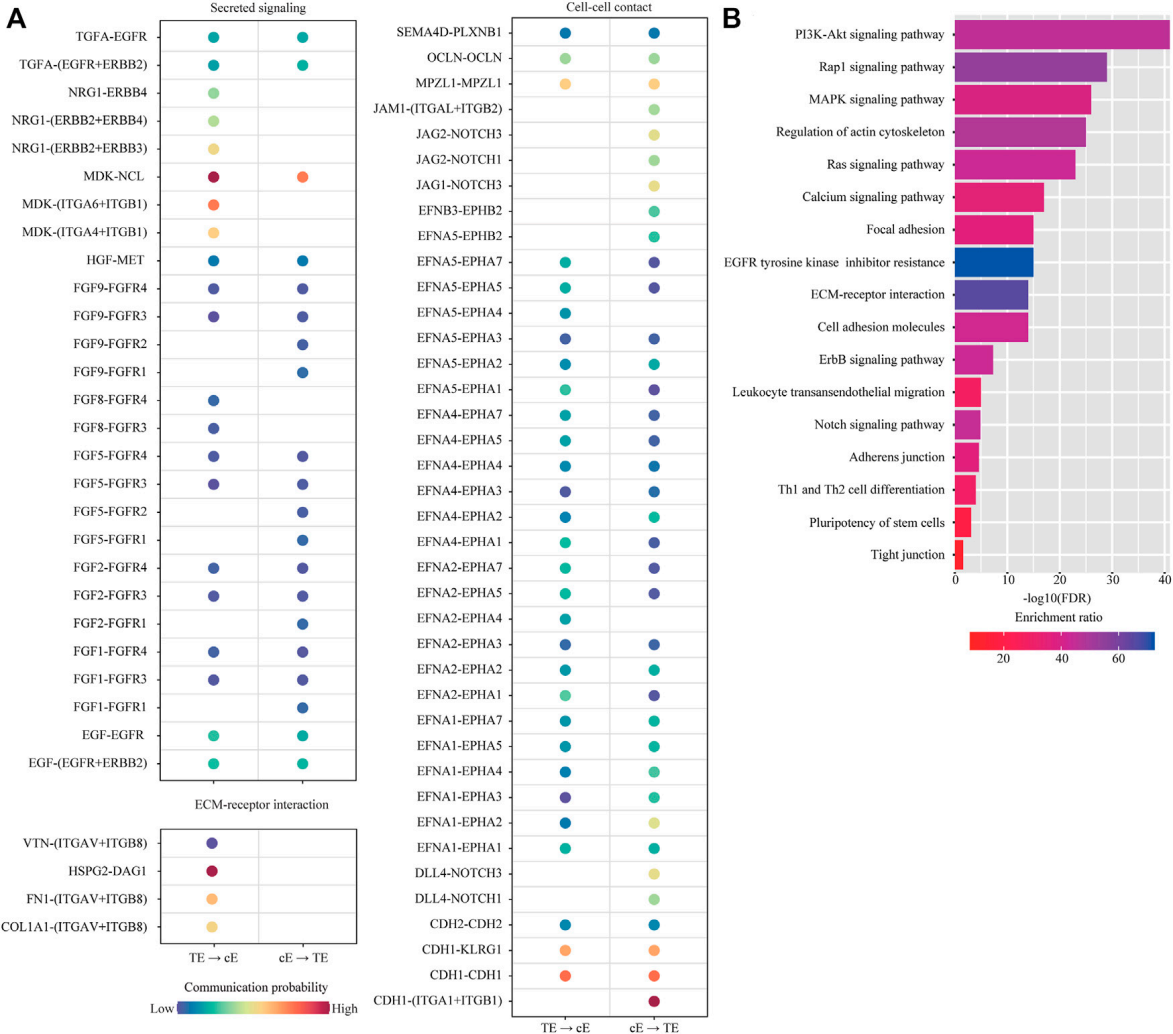
Cell-cell communication between ciliated epithelial cells and trophoblast cells. **(A)** Dot plot showing selected ligand-receptor interactions underlying the cross-talk between ciliated epithelial cells (cE) and trophoblast cells (TB). The communication probability defined by the CellChat software was indicated by color. **(B)** KEGG Pathway enrichment analysis of ligand-receptor pairs was performed by using the Metascape online tools.

Among them, we were particularly interested in the interactions between trophoblast cells (TB) and uterine epithelial cells (uE and cE), which represent the key mechanism of embryo implantation. In cell-cell communication analysis between TB and uE, we identified a total of 85 ligand-receptor interaction pairs (Figure 5A). Pathway analysis revealed that these ligand-receptor interactions were enriched among PI3K-Akt signaling pathway (FDR = 1.0 × 10^−48^), MAPK signaling pathway (FDR = 1.0 × 10^−41^), Ras signaling pathway (FDR = 1.0 × 10^−32^), Rap1 signaling pathway (FDR = 1.0 × 10^−26^), Calcium signaling pathway (FDR = 1.0 × 10^−23^), Regulation of actin cytoskeleton (FDR = 1.0 × 10^−20^), EGFR tyrosine kinase inhibitor resistance (FDR = 1.0 × 10^−14^), Focal adhesion (FDR = 1.0 × 10^−13^), ECM-receptor interaction (FDR = 1.0 × 10^−12^), Pluripotency of stem cells (FDR = 6.3 × 10^−9^), Cell adhesion molecules (FDR = 7.9 × 10^−9^), Cytokine-cytokine receptor interaction (FDR = 7.9 × 10^−9^), ErbB signaling pathway (FDR = 1.3 × 10^−7^), Neurotrophin signaling pathway (FDR = 3.2 × 10^−5^), Adherens junction (FDR = 6.3 × 10^−5^), JAK-STAT signaling pathway (FDR = 2.5 × 10^−3^), and TGF-beta signaling pathway (FDR = 5.0 × 10^−3^) (Figure 5B). In cell-cell communication analysis between TB and cE, we identified a total of 70 ligand-receptor interaction pairs (Figure 6A). Pathway analysis revealed that these ligand-receptor interactions were enriched among PI3K-Akt signaling pathway (FDR = 1.0 × 10^−41^), Rap1 signaling pathway (FDR = 1.0 × 10^−29^), MAPK signaling pathway (FDR = 1.0 × 10^−26^), Regulation of actin cytoskeleton (FDR = 1.0 × 10^−25^), Ras signaling pathway (FDR = 1.0 × 10^−23^), Calcium signaling pathway (FDR = 1.0 × 10^−17^), Focal adhesion (FDR = 1.0 × 10^−15^), EGFR tyrosine kinase inhibitor resistance (FDR = 1.0 × 10^−15^), ECM-receptor interaction (FDR = 1.0 × 10^−14^), Cell adhesion molecules (FDR = 1.0 × 10^−14^), ErbB signaling pathway (FDR = 5.0 × 10^−8^), Leukocyte transendothelial migration (FDR = 1.0 × 10^−5^), Notch signaling pathway (FDR = 1.3 × 10^−5^), Adherens junction (FDR = 2.5 × 10^−5^), Th1 and Th2 cell differentiation (FDR = 1.0 × 10^−4^), Pluripotency of stem cells (FDR = 7.9 × 10^−4^), and Tight junction (FDR = 2.5 × 10^−2^) (Figure 6B).

### 3.3 Cell Type Proportion Changes Upon Embryo Implantation

**FIGURE 7:**
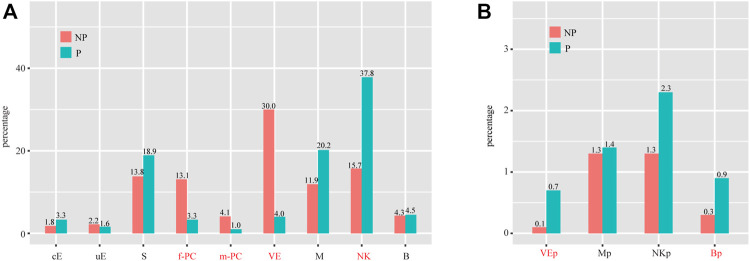
Cell population shifts in P compared to NP. **(A)** Bar plot showing the cell population changes of 11 major cell types with proliferating cells excluded. **(B)** Bar plot showing cell population changes of proliferating cells. Cell types with fold change (based on percentage) > 2 and *p*-value (χ^2^ test) < 0.05 were labeled in red. cE, ciliated epithelial cells; uE, unciliated epithelial cells; S, stromal cells; f-PC, fibroblast-like pericytes; m-PC, smooth muscle-like pericytes; VE, vascular endothelial cells; VEp, proliferating vascular endothelial cells; M, macrophages; Mp, proliferating macrophages; NK, natural killer cells; NKp, proliferating natural killer cells; B, B cells; Bp, proliferating B cells.

We investigated the abundance of each cell type in P compared to NP. By using the criteria of χ^2^ test *p* < 0.05 and fold change > 2, we found that the proportions of f-PC, m-PC and VE were significantly decreased, whereas the proportion of NK was significantly increased in P compared to NP (Figure 7A). Of special interest, we investigated the abundance of proliferating cells. We found that the proportions of Mp and NKp were unchanged, whereas the proportions of VEp and Bp were significantly increased (Figure 7B).

### 3.4 Cell Type Specific Transcriptional Changes for Embryo Implantation

**FIGURE 8:**
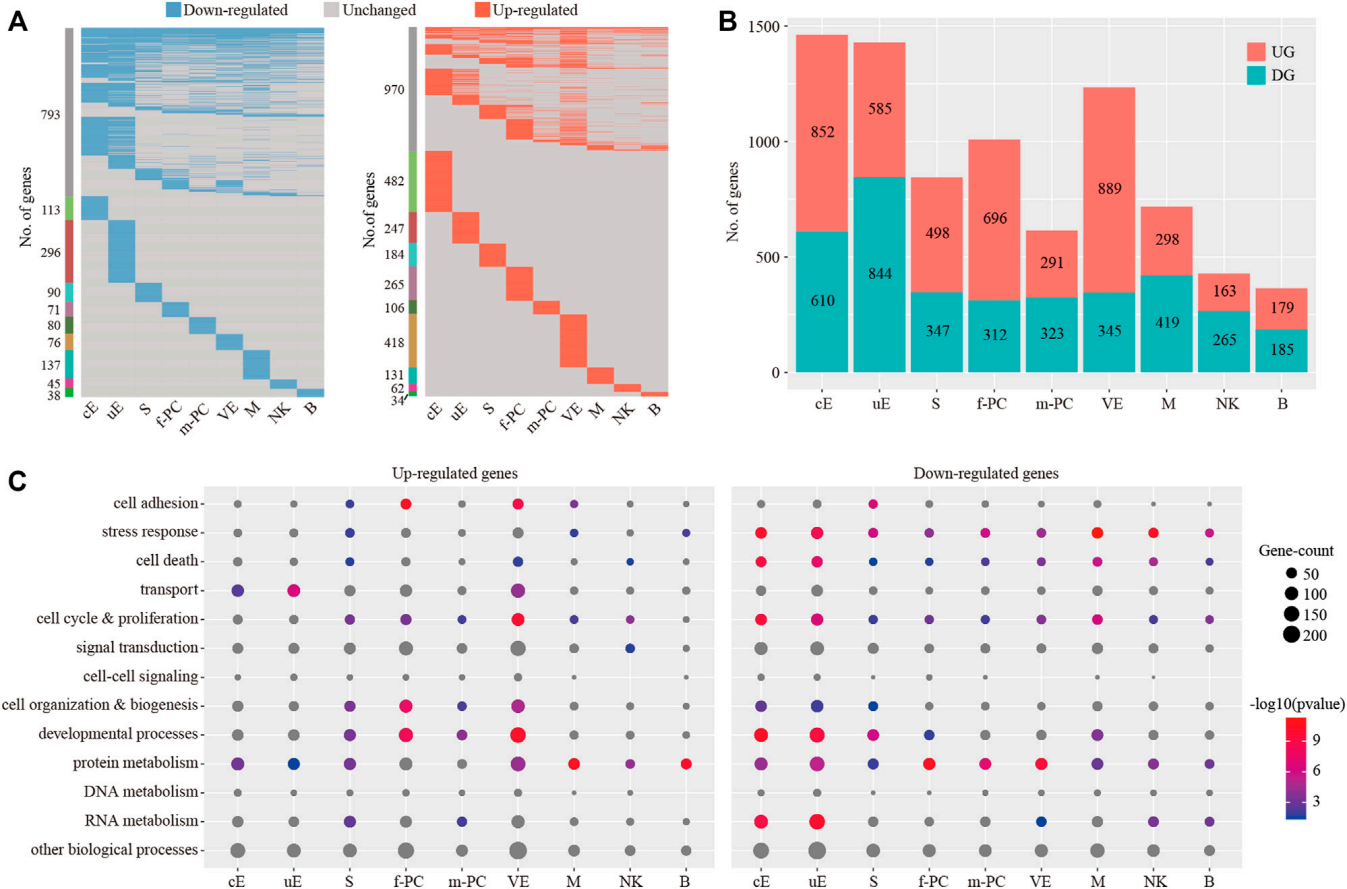
Identification of differentially expressed genes for each cell type. **(A)** Heatmap showing the distribution of differentially expressed genes (logFC > 0.25 and *p* < 0.05) in P compared to NP in each cell type. **(B)** The count of differentially expressed genes in each cell type. **(C)** Gene ontology (GO) enrichment analysis of down-regulated genes and up-regulated genes, respectively. *p* < 0.05 was used as the significance cutoff. Non-significant hits (*p* ≥ 0.05) were depicted in gray cE, ciliated epithelial cells; uE, unciliated epithelial cells; S, stromal cells; f-PC, fibroblast-like pericytes; m-PC, smooth muscle-like pericytes; VE, vascular endothelial cells; M, macrophages; NK, natural killer cells; B, B cells.

We investigated the breadth of transcriptional changes in each cell type by performing differential gene expression analysis (Figure 8A). Using a logFC cutoff of 0.25 and a pvalue cutoff of 0.05, we identified 1,462, 1,429, 845, 1,008, 614, 1,234, 717, 428 and 364 differentially expressed genes for cE, uE, S, f-PC, m-PC, VE, M, NK and B, respectively (Figure 8B and Supplementary Table S2). We then explored the biological implications of differentially expressed genes using gene ontology (GO) analysis. Enriched GO terms were provided in Figure 8C. These data indicated that each cell type invoked distinct biological processes to participate in embryo implantation.

### 3.5 Validation by Using Laser Capture Microdissection (LCM)-Coupled RNA-Seq Data

**FIGURE 9:**
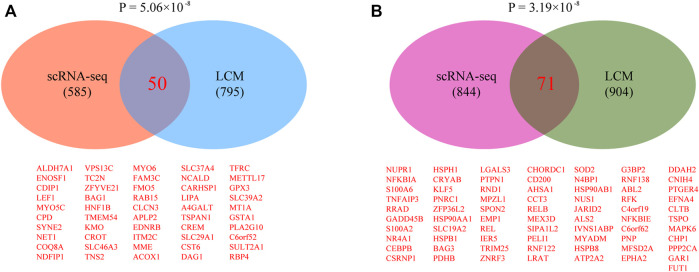
Validating single-cell RNA-seq data by laser capture microdissection (LCM)-coupled RNA-seq data. **(A)** Venn diagram showing the overlap of up-regulated genes. **(B)** Venn diagram showing the overlap of down-regulated genes. Raw LCM-coupled RNA-seq data were downloaded from the SRA database under accession number PRJNA668716. Differentially expressed genes were identified using a fold change of 2 and an adjusted *p*-value cutoff of 0.05 as described in the published paper (Int J Mol Sci. 2021 January 27; 22:1,248).

Previously, RNA-seq analysis was conducted on epithelial cells isolated by LCM from pig uterus on days 12 and 15 of pregnancy (Wang et al., 2021). Only mesometrial-side epithelial cell RNA-seq data were considered, since the attachment of pig conceptus to the endometrium normally takes place at the mesometrial side rather than the anti-mesometrial side (Dantzer 1985; Kridli et al., 2016). By comparing with our data, we identified 50 overlapped up-regulated genes (Figure 9A) and 71 overlapped down-regulated genes (Figure 9B), respectively. Notably, *p* < 0.05 was reached for all comparisons, providing validity of our single-cell RNA-seq data.

## 4 Discussion

The embryo implantation period is a crucial for pig reproduction. Here, we profiled the single-cell transcriptome for 12,415 cells from porcine endometrium and conceptus during embryo implantation. To the best of our knowledge, the present study is the first to highlight the transcriptome landscape associated with embryo implantation in pigs at single-cell resolution.

Previously, we performed single-cell RNA analysis of the mouse embryo implantation site on day 5 of pregnancy by using a whole uterine segment with an embryo in it (Yang et al., 2021a). However, it turned out that there was no trace of embryo-derived cell clusters in the data. An embryo is typically of no more than 100 cells. Of all the approximately 0.5 million cells obtained in the single-cell dissociation procedure, around 5000 cells could be sequenced per sample by the 10x platform. By calculating the probability, it seemed that only 1 cell per embryo could be captured in the single-cell RNA-seq data. The small number of embryonic cells within the uterine segment was a major problem (Yang et al., 2021a; He et al., 2021).

Therefore, in this study, instead of the whole uterus, we collected the embryo and a small amount of surrounding endometrial tissues at the pig implantation site for single-cell RNA-seq. The challenge of this study was to dissect embryonic cells from maternal endometrial cells in our data. This problem was solved in a multiple-step way. Firstly, using non-pregnant uterus as control, we could easily locate cell clusters that were unique for pregnant uterus. These cell clusters were potential embryonic cells. Secondly, embryonic cells and maternal endometrial cells are genetically different and many single-nucleotide polymorphisms (SNPs) might be captured by single-cell RNA-seq. We employed the Souporcell software (Heaton et al., 2020) to dissect embryonic cells from maternal uterine cells based on SNPs. In this way, we confirmed that the cell clusters that we identified in the previous step were indeed embryonic cells. Thirdly, we calculated signature genes for embryonic cells (i.e. genes expressed significantly higher in embryonic cell types than the maternal uterine cell types) by Wilcoxon rank-sum test. We found 2 embryonic cell types, erythroid cells and trophoblast cells. As expected, trophoblast cells, which are epithelial cells in nature, expressed KRT8 and KRT18 as signature genes. Notably, although the standard red blood cell lysis procedure was done before single-cell RNA-seq, it seemed that erythroid cells were resistant to this procedure. Finally, we used published datasets to validate our findings. Bulk-tissue RNA-seq was performed on Yorkshire pig embryos (Zang et al., 2021a) and endometrial tissues (unpublished) from days 9, 12 and 15 of pregnancy. Based on these data, we confirmed that HBE1 and HBZ were uniquely expressed in erythroid cells, while PEG10 was uniquely expressed in trophoblast cells. So far, no marker genes for pig trophoblast cells have been reported; thus, PEG10 deserves further investigation.

In pigs, to prepare for implantation, the blastocyst becomes ovoid and then tubular before rapidly elongating into a filamentous shape (Liu et al., 2018). This morphological change is believed to maximize contact between the embryo and the uterine surface. It has long been proposed that conceptus production of interleukin 1 beta 2 (IL1B2), estrogen (E_2_), prostaglandin E2 (PGE2) and interferon gamma (IFNG) are critical for conceptus development and implantation. Recently, CRISPR-Cas9 gene-editing technology provides an easy way to test these hypothesis by performing loss-of-function studies in the pig conceptus (Geisert et al., 2021). Inactivation of IL1B2 resulted in failure of rapid conceptus elongation (Whyte et al., 2018). By ablation of CYP19A1, it was proved that conceptus E_2_ is not essential for pre-implantation development and conceptus elongation, but is essential for maintenance of pregnancy (Meyer et al., 2019), likely by affecting transcription profile of the endometrium (Kaczynski et al., 2020). Interestingly, PTGS2 null conceptus was able to live beyond 30 days of gestation, suggesting that conceptus PGE2 is not essential for early pregnancy (Pfeiffer et al., 2020), probably because of a compensatory mechanism involving PGE2 auto-amplification loop in the endometrium in response to conceptus E_2_ (Waclawik et al., 2009). On the contrast, conceptus IFNG production is essential for endometrial proinflammatory response and conceptus attachment (Johns et al., 2021). The interaction between trophoblast cells and uterine epithelial cells represents the key mechanism of embryo implantation. In this study, we found 2 epithelial cell clusters, ciliated epithelial cells (cE, EPCAM^+^FOXJ1^+^) and unciliated epithelial cells (uE, EPCAM^+^FOXJ1^-^). This result was in line with the human endometrium (Wang et al., 2020), but was different from the mouse endometrium (Yang et al., 2021b). According to the expression pattern of glandular cell marker FOXA2, it seemed that both cE and uE contained luminal and glandular epithelial cells. However, due to the dropout effect we mentioned before (Yang et al., 2021b), it was unsafe to dissect luminal and glandular epithelium from cE and uE based on FOXA2 expression (i.e. FOXA2 = 0 luminal epithelium and FOXA2>0 glandular epithelium). Therefore, we were unable to label luminal and glandular epithelial cells in this study. The unique markers for luminal and glandular epithelial cells in pig endometrium are yet to be discovered. By examining the secreted signaling, we found that the interaction between TB and uE was mediated by secreted proteins TGFA, NTF3/5, NRG1, MDK, INHBB, INHBABB, IL1A, IL10, HGF, FGF1/2/5/8/9/10, EGF and BDNF. Interestingly, a majority of these proteins were expressed in both TB and uE. Additionally, we found that ITVAV and ITGB8 expressed on uE were key mediators for ECM-receptor interaction. Apart from secreted singling and ECM-receptor interaction, TB might also cross-talk with uE by cell-cell contact via homophilic OCLN, MPZL1, CDH1 and CDH2, as well as via the bipartite ephrin ligand/Eph receptor pathway. Similar results were found between TB and cE. Global intercellular cross-talk between all cell types was provided in Supplementary Table S3. Our data provide clues for the molecular mechanism underlying implantation from the aspect of cell-cell communication.

The blastocyst-uterine interaction is a trigger for endometrial changes upon implantation. In this study, we found that the cell type composition for P was 13.9% stromal cells, 3.6% epithelial cells, 3.1% pericytes, 3.4% endothelial cells, 49.2% immune cells, and 26.7% fetal cells, while the cell type composition for NP was 13.8% stromal cells, 4.0% epithelial cells, 17.2% pericytes, 30.1% endothelial cells, and 34.8% immune cells. We used 2 mg/ml Collagenase II and 10 mg/ml Dispase II for single-cell suspension preparation. Collagenase II is a crude collagenase preparation with weak trypsin-like activity. Because trypsin might cause damage to cells and disturb gene expression (van den Brink et al., 2017), it was not used in this study. Previously, by using histologic and morphometric analysis, it was estimated that there are 47% stromal cells, 37% luminal epithelial cells and 16% glandular epithelial cells in the pig endometrium (Blackwell et al., 2003). We carefully examined our raw data (unfiltered data) and found many more epithelial cells. However, after quality control (See Materials and Methods), most of these epithelial cells were discarded. The discarded epithelial cells were regarded as “dying cells”. This might be the reason why less epithelial cells were found than expected in our scRNA-seq data. Of note, the estimated percentages for each cell type may be distorted from their actual proportions in the pig endometrium, as the recovery rate for each cell type might vary during the cell dissociation procedure. Moreover, the NP sample was randomly collected from the mesometrial side of pig endometrium, which was not a strict control sample for P. Therefore, the changes in cell type composition between P and NP might not reflect a real biological effect. A better design for tissue collection is needed in the future.

We investigated the breadth of transcriptional changes for each cell type in P compared to NP by performing differential gene expression analysis. As expected, the epithelial cells (cE and uE) had the largest number of differentially expressed genes. Gene ontology analysis revealed that transport and protein metabolism were significantly enriched among up-regulated genes, while stress response, cell death, cell cycle and proliferation, cell organization and biogenesis, developmental processes, protein metabolism and RNA metabolism were significantly enriched among down-regulated genes. We identified 845 differentially expressed genes in stromal cells (S), of which 498 genes were up-regulated and 347 genes were down-regulated in P compared to NP. Gene ontology analysis showed that cell adhesion, stress response, cell death, cell cycle and proliferation, cell organization and biogenesis, developmental processes, protein metabolism and RNA metabolism were significantly enriched among up-regulated genes, while cell adhesion, stress response, cell death, cell cycle and proliferation, cell organization and biogenesis, developmental processes and protein metabolism were significantly enriched among down-regulated genes. In pericytes (f-PC and m-PC), gene ontology terms significantly enriched among up-regulated genes were cell adhesion, cell cycle and proliferation, cell organization and biogenesis, developmental processes and RNA metabolism, and gene ontology terms significantly enriched among down-regulated genes were stress response, cell death, cell cycle and proliferation, developmental processes and protein metabolism. There were 889 up-regulated genes and 345 down-regulated genes in vascular endothelium (VE). Based on gene ontology, cell adhesion, cell death, transport, cell cycle and proliferation, cell organization and biogenesis, developmental processes and protein metabolism were significantly enriched among up-regulated genes, while stress response, cell death, cell cycle and proliferation, protein metabolism and RNA metabolism were significantly enriched among down-regulated genes. For immune cells (M, NK and B), gene ontology terms significantly enriched among up-regulated genes were cell adhesion, stress response, cell death, cell cycle and proliferation, signal transduction and protein metabolism, and gene ontology terms significantly enriched among down-regulated genes were stress response, cell death, cell cycle and proliferation, developmental processes, protein metabolism and RNA metabolism.

In conclusion, this study provided a comprehensive single-cell transcriptome atlas for porcine conceptus and endometrium during embryo implantation. Our data present a valuable resource for deciphering the molecular mechanism underlying embryo implantation in pigs.

## Data Availability

The datasets presented in this study can be found in online repositories. The names of the repository/repositories and accession number(s) can be found in the article/Supplementary Material.
